# The Atlanta Medical Society

**Published:** 1882-11

**Authors:** 


### Societies.

### THE ATLANTA MEDICAL SOCIETY.

*Reported for the Atlanta Medical Rejister.*

ATLANTA, September, 1882.

Dr. E. L. Connally in the chair.

Dr. John G. Earnest, the regularly appointed leader of debate for the evening, read an essay on Pelvic Cellulitis. See page 65.

Dr. W. D. Bizzell referred to the interesting paper just read, and in discussing the essay, stated that the effect produced by inflammation in the cellular structures of the pelvis, even after all active inflammation had subsided and a good portion of the inflammatory deposit had been absorbed, was not to be lost sight of; that with each recurrence of the menstrual molimen a great deal of irritation might be developed, and even a localized oophoritis, and when these were not developed the nerves of the part seemed to remain in a morbidly sensitive condition, and violent pains of a neuralgic character would be developed after the least exertion, as walking, riding, etc. In this class of cases he had found the prolonged use of electricity of great use indeed. Applied by means of a copper plate, on which the patient placed the bare feet, and by means of a sponge applied to the spine, the abdomen and the lower half of the body, a considerable current, fine and rapidly interrupted, of the Faradic order, was allowed to flow through this part of the body. The effect was that the patient was enabled to take more exercise, and tonics and proper feeding seemed to act better, and by two seances of one half hour duration, twice a day, for two or three months, the patient was slowly restored from a state of bedridden invalidism to almost perfect health. He thought that morbid conductivity and irritability which dominates the patient in this class of cases was too often lost sight of by the physician, and electricity, together with the hot water injections so earnestly commended by Dr. Emmett, would restore many women to society who are now classed as invalids.

Dr. F. H. O'Brien alluded to the possible value of Battey's operation in cases of cellulitis terminating in ovarian displacement and resulting disease.

Dr. Earnest, in reply to a question, said his experience had taught him that adhesions were more troublesome than Dr. Emmett seems to think them. He recalls two cases in which, by the use of proper pessaries, he succeeded in elevating the uterus to a certain extent, but not to its normal position. The adhesions could only be partially overcome.

His treatment, in a word, consists in subduing inflammation by hot water injections, rest in bed, etc., and then, after tolerance is established, introducing a Hodge's pessary, and substituting, by slow degrees, others having greater curves, which are calculated to exert firmer pressure.

An important point in these cases is the derangement of the general circulation, due to irregular action of the heart, which is dependent upon retroversion of the womb. This assertion may seem strange to some, but he has satisfied himself of the intimate connection between the two conditions. As the adhesions are stretched and the malposition corrected, the abnormal heart's action subsides and the frequently disordered digestion is improved.

Dr. Baird remarked that the pain incident to defecation and the constipating effects of appropriate drugs favored an accumulation of hardened fæces in the rectum. In one case recently under his care this was a source of much suffering, which was only relieved after the removal of the marble-like masses by means of the finger, well oiled and gently introduced and manipulated. He regards this a practical point of importance in connection with acute pelvic cellulitis.

Dr. E. L. Connally said he now had under treatment a a patient who imprudently leaped from the back of a horse to the ground, last year, and an attack of pelvic cellulitis resulted. She had previously suffered greatly from dysmenorrhœa. She was confined to her bed for four months. Her temperature ranged from 101° to 103°. He prescribed hot water douches, rest, anodynes, etc. The adhesions which formed have fixed the uterus in the axis of the vagina. He is now using pressure by means of the cotton vaginal tampon. Thinks there is some improvement, but it is very slow. The general health of the lady is better. He referred, incidentally, to a case of periuterine hematocele seen several years ago, which was opened through the vagina, giving vent to a large quantity of bloody pus.

